# Identification of potential miRNA–mRNA regulatory network contributing to pathogenesis of HBV-related HCC

**DOI:** 10.1186/s12967-018-1761-7

**Published:** 2019-01-03

**Authors:** Weiyang Lou, Jingxing Liu, Bisha Ding, Danni Chen, Liang Xu, Jun Ding, Donghai Jiang, Lin Zhou, Shusen Zheng, Weimin Fan

**Affiliations:** 1 Program of Innovative Cancer Therapeutics, Division of Hepatobiliary and Pancreatic Surgery, Department of Surgery, First Affiliated Hospital, College of Medicine, Key Laboratory of Organ Transplantation, Zhejiang University, 79 Qingchun Road, Hangzhou, 310003 Zhejiang Province China; 2 Key Laboratory of Organ Transplantation, Hangzhou, 310003 Zhejiang Province China; 3 Key Laboratory of Combined Multi-organ Transplantation, Ministry of Public Health, Hangzhou, 310000 China; 4 Department of Intensive Care Unit, Changxing People’s Hospital of Zhejiang, Huzhou, 313100 Zhejiang Province China; 5 Department of Pathology and Laboratory Medicine, Medical University of South Carolina, Charleston, SC 29425 USA

**Keywords:** MicroRNAs (miRNAs), Hepatocellular carcinoma (HCC), Hepatitis B virus (HBV), Bioinformatic analysis, Kaplan–Meier plotter (KM-plotter)

## Abstract

**Background:**

Hepatitis B virus (HBV) is one of the major risk factors of hepatocellular carcinoma (HCC). Increasing evidence indicates that microRNA (miRNA)–mRNA axis is involved in HCC. However, a comprehensive miRNA–mRNA regulatory network in HBV-related HCC is still absent. This study aims to identify potential miRNA–mRNA regulatory pathways contributing to pathogenesis of HBV-related HCC.

**Methods:**

Microarray GSE69580 was downloaded from Gene Expression Omnibus (GEO) database. GEO2R and ‘R-limma’ were used to conduct differential expression analysis. The common miRNAs appeared in the two analytic sets were screened as potential differentially expressed miRNAs (DE-miRNAs). The prognostic roles of screened DE-miRNAs in HCC were further evaluated using Kaplan–Meier plotter database. Target genes of DE-miRNAs were predicted by miRNet. Then, protein–protein interaction (PPI) networks were established for these targets via the STRING database, after which hub genes in the networks were identified by Cytoscape. Functional annotation and pathway enrichment analyses for the target genes were performed through DAVID database. Three enriched pathways related to HBV-related HCC were selected for further analysis and potential target genes commonly appeared in all three pathways were screened. Cytoscape was employed to construct miRNA-hub gene network. The expression and correlation of potential miRNAs and targets were further detected in clinical HBV-related HCC samples by qRT-PCR.

**Results:**

7 upregulated and 9 downregulated DE-miRNAs were accessed. 5 of 7 upregulated DE-miRNAs and 5 of 7 downregulated DE-miRNAs indicated significant prognostic roles in HCC. 2312 and 1175 target genes were predicted for the upregulated and downregulated DE-miRNAs, respectively. TP53 was identified as the hub gene in the PPI networks. Pathway enrichment analysis suggested that these predicted targets were linked to hepatitis B, pathways in cancer, microRNAs in cancer and viral carcinogenesis. Further analysis of these pathways screened 20 and 16 target genes for upregulated and downregulated DE-miRNAs, respectively. By detecting the expression of 36 target genes, six candidate target genes were identified. Finally, a potential miRNA–mRNA regulatory network was established based on the results of qRT-PCR and expression correlation analysis.

**Conclusions:**

In the study, potential miRNA–mRNA regulatory pathways were identified, exploring the underlying pathogenesis and effective therapy strategy of HBV-related HCC.

**Electronic supplementary material:**

The online version of this article (10.1186/s12967-018-1761-7) contains supplementary material, which is available to authorized users.

## Background

As a major public health issue, hepatocellular carcinoma (HCC) is the fifth most common malignancy in men and the second leading cause of cancer-related mortality globally [1]. HCC is also the most common primary liver cancer, approximately representing 90% of primary liver cancer cases [2].

Multiple risk factors are linked to the onset and progression of HCC, among which hepatitis B virus (HBV) infection is one of the leading risk factors [3]. Reported by World Health Organization, in 2015, 257 million people were infected with HBV, and deaths caused by HBV were 0.78 million [4]. To date, it has been well documented that the development of HBV-related HCC is a three-stage process: liver inflammation-liver cirrhosis-HCC [3]. However, the underlying molecular mechanisms of HBV-related HCC remains largely insufficient and need to be further deeply elucidated.

MicroRNAs (miRNAs) are a group of endogenous single-strand non-coding RNA molecules that approximately contain 19–25 nucleotides [5]. MiRNAs are found to function as key post-transcriptional regulators in a variety of cellular biological processes, such as differentiation, proliferation, apoptosis, migration and invasion [6]. Over the past few decades, mounting studies have suggested that miRNAs can act as oncogenes or tumor suppressors in most types of cancer, including breast cancer [7], gastric cancer [8], prostate cancer [9] and colorectal cancer [10]. With regard to HCC, multiple previous studies have also demonstrated that miRNAs are involved in HCC progression [11–13]. However, only few reports regarding the roles of miRNAs in HBV-related HCC. The team of Xiaojie Xu found that HBV X protein (HBx), a virally encoded protein exerting a crucial role in the pathogenesis of HBV-related HCC, inhibited miR-148a to fuel carcinogenesis of HCC [14]. MiR-7/21/107 were also shown to contribute to HBx-induced HCC progression [15]. MiR-338-3p was demonstrated to be involved in HBV preS2-induced HCC tumorigenesis by upregulation of TAZ [16]. To our knowledge, there is no systemic and comprehensive analysis of miRNA–mRNA regulatory network in tumorigenesis of HBV-related HCC.

Combination of high-throughput technologies and bioinformatic analysis can provide researchers and scholars with unprecedent convenience in seeking novel cancer biomarkers and therapeutic targets. In this study, the miRNA expression profile from five HBV-related HCC patients was first downloaded from Gene Expression Omnibus (GEO, GSE69580). Then, we successively conducted differential expression analysis, target gene prediction, GO functional annotation, KEGG enrichment, analysis of potential target genes and construction of potential miRNA–mRNA pathways. To further validate these potential miRNA–mRNA pathways in HBV-related HCC, qRT-PCR were performed to detect the expression levels of these miRNAs and targets in HBV-related HCC clinical samples, and the correlation of miRNA and mRNA expression was finally evaluated.

## Materials and methods

### MicroRNA microarray

In the course of seeking relative datasets, we only included studies regarding comparing the miRNA expression in HBV-related HCC tissue with normal liver tissue from the Gene Expression Omnibus (GEO) database (http://www.ncbi.nlm.nih.gov/geo). Only datasets regarding clinical tissues were collected, and those datasets that studied mRNA expression in the level of cell line were excluded. The information of collected datasets containing titles and abstracts was screened. Then, those datasets of interest were further assessed. In the end, only the dataset, GSE69580 based on the platform of GPL10850 Agilent-021827 Human miRNA Microarray (V3) (miRbase release 12.0 miRNA ID version), was met the criterion and was chosen for subsequent analysis. This dataset contained five HBV-related HCC tumor tissues and five non-tumor liver tissues.

### Screening of candidate DE-miRNAs

To screen differentially expressed miRNAs (DE-miRNAs) between HBV-related HCC tumor tissue and non-tumor liver tissue, two methods of differential expression analysis were successively conducted. GEO2R analytic tool provided by GEO database [17] was first applied to screen DE-miRNAs. Besides, another method named “R-limma” was introduced to further screen DE-miRNAs. Firstly, Series Matrix File of GSE69580 was downloaded from the GEO database. Data were then normalized using the normalizeBetweenArray function from R package ‘LIMMA’ from the Bioconductor project [18]. Data before and after normalization were presented in Additional file 1: Figure S1. Subsequently, the differential analysis was conducted by the LIMMA package through entering related codes into R (version 3.4.4). The *P* value < 0.01 and |log_2_FC| > 2 were set as the cut-off criterion in the two methods mentioned above. The common DE-miRNAs identified by the two methods were presented as Veen diagram and identified as candidate DE-miRNAs.

### Survival analysis of candidate DE-miRNAs

The Kaplan–Meier plotter (KM plotter) database, an online database established using gene expression data and survival information of cancer patients downloaded from the GEO database, was utilized to analyze the prognostic roles of screened candidate DE-miRNAs in HCC [19]. Briefly, these miRNAs were first typed into the database. Subsequently, KM survival plots were created, and hazard ratio (HR), 95% confidence interval (CI) and log rank *P*-value were displayed on the webpage. Log rank *P*-value < 0.05 was considered as statistically significant. Only high expression of upregulated and downregulated candidate DE-miRNAs indicating an unfavorable and favorable prognosis in patients with HCC were selected for further analysis, respectively. These selected DE-miRNAs were named as potential DE-miRNAs.

### Prediction and analysis of target genes of potential DE-miRNAs

The target genes of potential DE-miRNAs were predicted using miRNet (http://www.mirnet.ca/), which is an easy-to-use comprehensive tool integrated data from eleven different miRNA databases (TarBase, miRTarBase, miRecords, miRanda, miR2Disease, HMDD, PhenomiR, SM2miR, PharmacomiR, EpimiR and starBase) [20]. Then, protein–protein interaction (PPI) network of target genes was constructed using the STRING database (http://string-db.org) [21]. Subsequently, the hub genes in the network were identified by analyzing the degree of connectivity using Cytoscape software (version 3.6.1) [22].

### Functional annotation and pathway enrichment analysis

The online program, the Database for Annotation, Visualization and Integrated Discovery (DAVID, http://david.abcc.ncifcrf.gov/), is a comprehensive tool for researchers and scholars to understand biological meaning behind multiple genes [23]. In this study, DAVID was used to perform Gene Ontology (GO) functional annotation and Kyoto Encyclopedia of Genes and Genomes (KEGG) pathway enrichment analysis for predicted target genes of potential DE-miRNAs. *P*-value < 0.05 was regarded as statistically significant.

### Screening of genes in HBV-related HCC

This study aims to identify miRNA–mRNA regulatory pathways associated with HBV-related HCC. Therefore, after conducting KEGG pathway enrichment analysis, several HBV-related HCC pathways were selected for further analysis. For upregulated potential DE-miRNAs, three enriched pathways including hsa05161, hsa05200 and hsa05203 were screened. For downregulated potential DE-miRNAs, three enriched pathways including hsa05161, hsa05200 and hsa05206 were screened. The same genes presented in all three pathways were further chosen to study. These selected target genes were named as candidate target genes.

### Construction of miRNA-hub gene network

To explore the correlation of candidate target genes with potential DE-miRNAs, Cytoscape software (version 3.6.1) was employed to construct and analyzed the miRNA-hub gene network.

### Analysis of candidate target gene expression using the Oncomine and UALCAN database

Two databases, Oncomine database (http://www.oncomine.org) [24] and UALCAN database (http://ualcan.path.uab.edu/index.html) [25], were introduced to analyze the expression of these candidate target genes. Firstly, all candidate target genes were entered into the Oncomine database, which is an online cancer microarray database. *P*-value < 1E−4, fold change > 2 and gene rank in top 10% were set as the cut-off criterion. Only target genes of upregulated DE-miRNAs were significantly downregulated while targets genes of downregulated DE-miRNAs were significantly upregulated in HCC were identified as potential target genes. The expression levels of these potential target genes in HCC were further validated using the UALCAN database, which is a user-friendly, interactive web resource for analyzing cancer transcriptome data. *P*-value < 0.05 was considered as statistically significant.

### Cell culture

The HBV-related HCC cell line, Hep3B, was obtained from the Institute of Biochemistry and Cell Biology of the Chinese Academy of Sciences (Shanghai, China). The cell line was maintained in Dulbecco’s modified Eagle’s medium (DMEM; Gibco, 12430047) containing 10% fetal bovine serum (FBS; Biological Industries, 04-0101-1, Cromwell, CT, USA), 100 mg/mL streptomycin, and 100 U/mL penicillin (Invitrogen, Shanghai, China) under a humidified atmosphere of 5% CO_2_ at 37 °C.

### Clinical tissues

Fresh frozen HBV-related HCC clinical tissues and matched adjacent normal liver tissues were obtained from 20 patients who had undergo surgery from 2016 to 2017 at the First Affiliated Hospital of Zhejiang University School of Medicine (Hangzhou, China). Tissues were frozen and stored in liquid nitrogen. The study was approved by the Ethics Committee of the First Affiliated Hospital of Zhejiang University School of Medicine, and written informed consent from each patient was obtained.

### Transfection of cell line

Hep3B cells were seeded in six-well plates, after which the miRNA mimics (50 nM) and negative control (50 nM) were transfected into the cell line by Lipofectamine™ 3000 (Invitrogen, Shanghai, China) according to the manufacturer’s instruction. The miRNA mimics and mimic NC were purchased from RiboBio Co. Ltd (Guangzhou, China).

### qRT-PCR

The expression of potential DE-miRNAs and target genes in clinical samples were analyzed by qRT-PCR as previously reported [26]. Firstly, total RNA was extracted from tissue samples using RNAiso plus Reagent (TaKaRa, Kusatsu, Japan). MiRNA reverse transcription primers and q-PCR primers were purchased from RiboBio Co. Ltd (Guangzhou, China), and mRNA q-PCR primers (Additional file 2: Table S1) were synthesized by BGI (Beijing, China). Then, total RNA was reverse-transcribed into complementary DNA (cDNA) by the PrimeScript RT Reagent Kit (TaKaRa, RR0037A). Next, q-PCR was performed in triplicates in a Roche LightCycler480 II Real-Time PCT Detection System by SYBR Premix Ex Taq (TaKaRa, RR420A). The expression level of miRNAs (relative to U6) and target genes (relative to GAPDH) were analyzed using the method of 2^−ddCt^.

### Statistical analysis

Experiments were performed in triplicates and the results were shown as mean ± standard deviation (SD). Statistical analysis was conducted using the GraphPad Prism Software (Version 7). The differences between two groups were estimated by a two-tailed Student’s *t*-test. Spearman correlation coefficients were calculated to evaluate the correlations.

## Results

### Candidate DE-miRNAs Identification and survival analysis

**Fig. 1:**
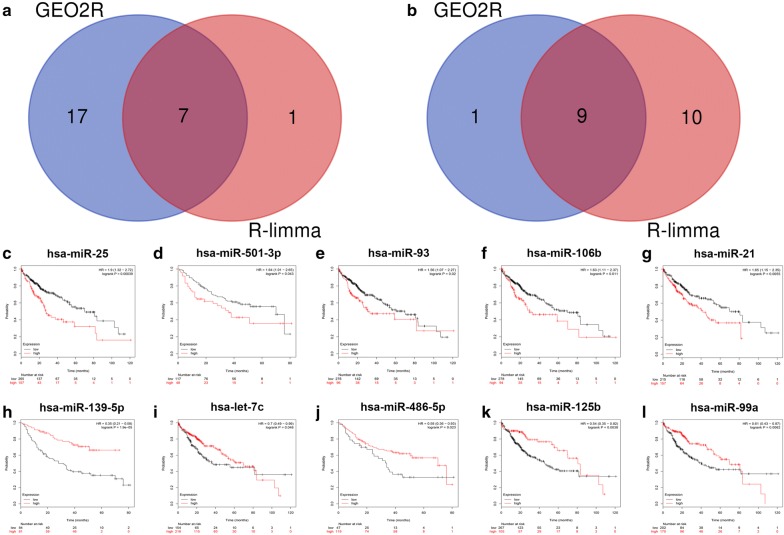
Identification of potential DE-miRNAs. **a**, **b** The intersection of two differential expression analytic methods, GEO2R and R-limma: **a** for upregulated DE-miRNAs; **b** for downregulated DE-miRNA. **c**–**l** The significant prognostic roles of potential DE-miRNAs in HCC: **c** for hsa-miR-25; **d** for hsa-miR-501-3p; **e** for hsa-miR-93; **f** for hsa-miR-106b; **g** for hsa-miR-21; **h** for hsa-miR-139-5p; **i** for hsa-let-7c; **j** for hsa-miR-486-5p; **k** for hsa-miR-125b; **l** for hsa-miR-99a

As mentioned above, two methods were employed to identify DE-miRNAs. The results of GEO2R analysis showed that there were 24 upregulated DE-miRNAs and 10 downregulated DE-miRNAs in HBV-related HCC tumor samples. There were eight upregulated DE-miRNAs and 19 downregulated DE-miRNAs in HBV-related HCC tumor samples after being compared with normal liver samples using ‘R-limma’ analytic method. Notably, as presented in Fig. 1a, b, seven upregulated DE-miRNAs and nine downregulated DE-miRNAs commonly appeared in the results of two analytic methods, so they were identified as candidate DE-miRNAs. These candidate DE-miRNAs were listed in Table 1.

**Table 1:** Common (GEO2R and R-limma) differentially expressed miRNAs (DE-miRNAs) between HBV-related HCC tumor versus non-tumor liver tissue

Upregulated DE-miRNA	log_2_FC	t	B	P value
hsa-miR-25-3p	1.86	5.2	− 0.0946	7.03E−04
hsa-miR-501-3p	7.01	19.1	8.4883	3.14E−08
hsa-miR-93-5p	1.84	3.66	− 2.2376	5.84E−03
hsa-miR-532-5p	5.83	3.46	− 2.5537	7.99E−03
hsa-miR-106b-5p	2.68	5.37	0.1219	5.67E−04
hsa-miR-21-5p	3.89	6.85	1.8157	1.03E−04
hsa-miR-151-3p	3.38	5.38	0.14	5.57E−04

Downregulated DE-miRNA	log_2_FC	t	B	P value
hsa-miR-199a-3p	− 3.06	− 3.85	− 1.9612	4.45E−03
hsa-miR-139-5p	− 3.48	− 5.58	0.3815	4.38E−04
hsa-miR-30a-3p	− 6.24	− 3.93	− 1.8413	3.95E−03
hsa-let-7c-5p	− 2.14	− 3.52	− 2.4572	7.26E−03
hsa-miR-486-5p	− 3.83	− 5.1	− 0.2182	7.95E−04
hsa-miR-125b-5p	− 3.43	− 6.63	1.5818	1.31E−04
hsa-miR-99a-5p	− 2.4	− 3.45	− 2.5639	8.07E−03
hsa-miR-10a-5p	− 1.37	− 3.83	− 1.9874	4.56E−03
hsa-miR-199a-5p	− 2.95	− 6.02	0.899	2.61E−04

To further validate these candidate DE-miRNAs, the prognostic roles of them in HCC were subsequently assessed by using KM plotter database. As shown in Fig. 1c–I, among 16 candidate DE-miRNAs, only high expression of five upregulated DE-miRNAs (miR-25-3p, miR-501-3p, miR-93-5p, miR-106b-5p and miR-21-5p) and five downregulated DE-miRNAs (miR-139-5p, let7c-5p, miR-486-5p, miR-125b-5p and miR-99a-5p) indicated a significantly unfavorable and favorable OS in patients with HCC, respectively. Therefore, these miRNAs were identified as final candidate DE-miRNAs (potential DE-miRNAs) and were selected for further analysis. None of expression of the other six candidate DE-miRNAs was significantly related to prognosis of patients with HCC.

### Target prediction and analysis of candidate DE-miRNAs

**Fig. 2:**
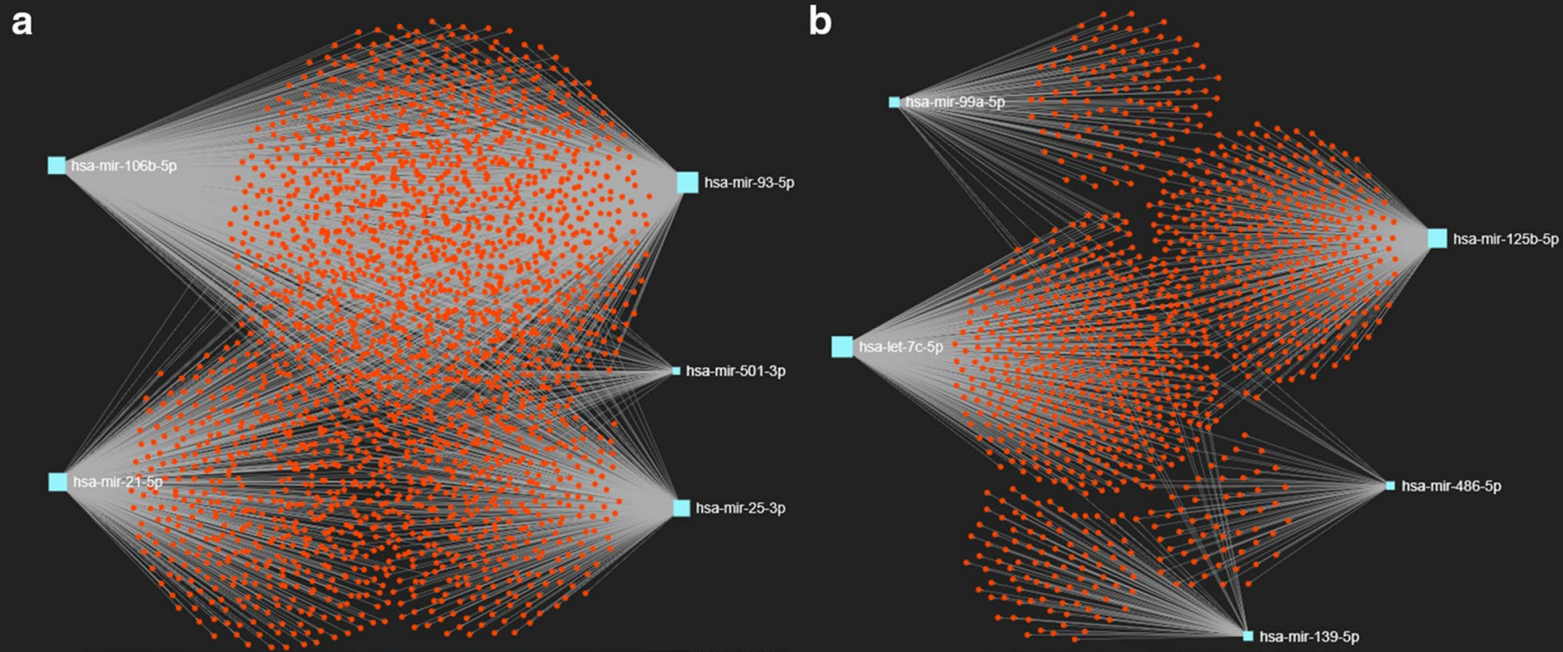
The predicted target genes of potential DE-miRNAs. **a** For upregulated DE-miRNAs; **b** for downregulated DE-miRNAs

The target genes of five potential upregulated and downregulated DE-miRNAs were successively predicted by miRNet. As shown in Table 2, we got total 2312 and 1175 predicted targets of the upregulated and downregulated DE-miRNAs, respectively. For the five upregulated DE-miRNAs, miR-93-5p was found to potentially target the most genes, with the number of 1220. For the five downregulated DE-miRNAs, let-7c-5p possessed the most targets, which number is 516. For better visualization, miRNA–mRNA networks were established using miRNet database as depicted in Fig. 2.

**Table 2:** The target number of the upregulated and downregulated DE-miRNAs

Upregulated DE-miRNA	Number	Downregulated DE-miRNA	Number
hsa-miR-93-5p	1220	hsa-let-7c-5p	516
hsa-miR-106b-5p	1091	hsa-miR-125b-5p	432
hsa-miR-21-5p	612	hsa-miR-99a-5p	133
hsa-miR-25-3p	518	hsa-miR-139-5p	105
hsa-miR-501-3p	69	hsa-miR-486-5p	67
Total	2312	Total	1175

**Fig. 3:**
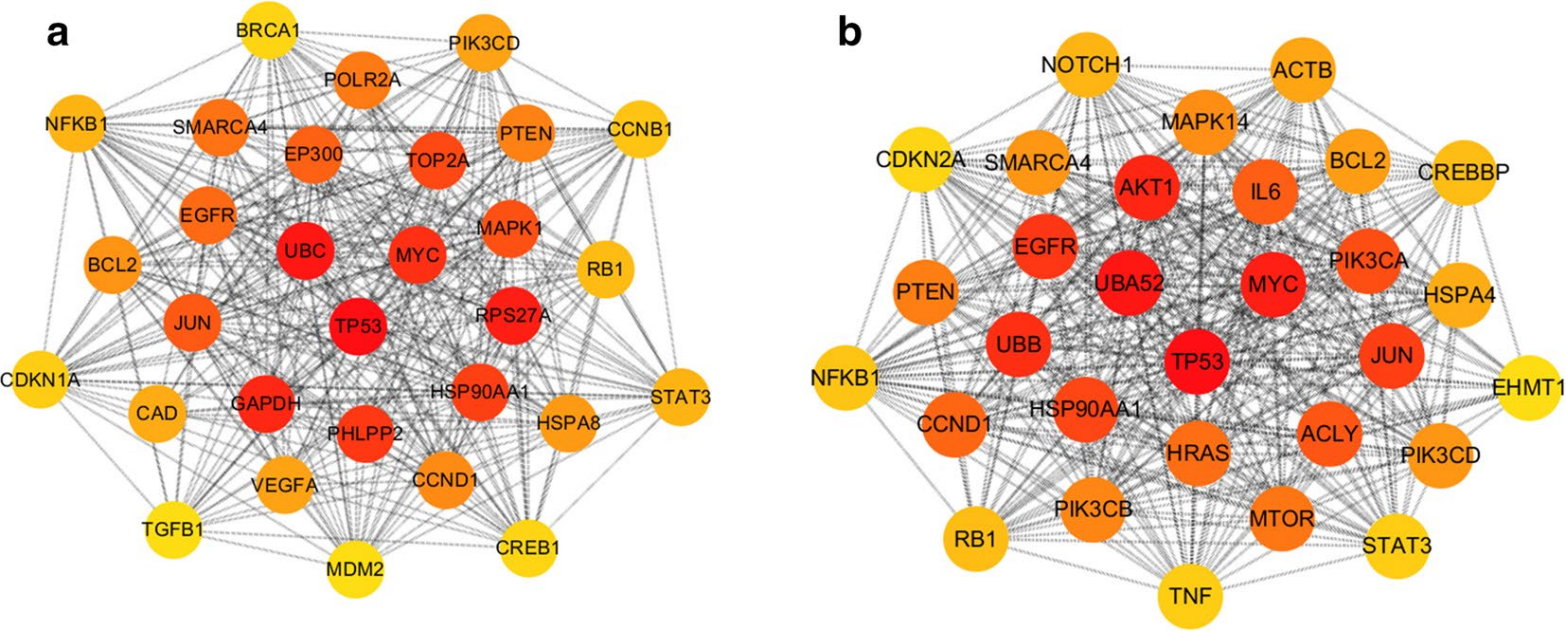
The top 30 hub genes in protein–protein interaction (PPI) network of predicted target genes. **a** For upregulated DE-miRNAs; **b** for downregulated DE-miRNAs

Next, PPI networks of predicted target genes of five upregulated DE-miRNAs (Fig. 3a) and five downregulated DE-miRNAs (Fig. 3b) were separately constructed using the STRING database and Cytoscape software. According to degree, the top 10 hub genes in the networks were screened out and were listed in Table 3. For upregulated DE-miRNAs, the top 10 hub genes were *TP53, UBC, RPS27A, GAPDH, MYC, PHLPP2, HSP90AA1, TOP2A, MAPK1* and *JUN*. For downregulated DE-miRNAs, the top 10 hub genes were *TP53, UBA52, MYC, AKT1, UBB, EGFR, JUN, HSP90AA1, PIK3CA* and *ACLY*. TP53 showed the highest node degree in both two sets, which were 336 and 248, respectively.

**Table 3:** Hub genes identified in the PPI networks

Upregulated DE-miRNAs		Downregulated DE-miRNAs	
Gene symbol	Degree	Gene symbol	Degree
TP53	336	TP53	248
UBC	274	UBA52	193
RPS27A	255	MYC	177
GAPDH	249	AKT1	165
MYC	223	UBB	163
PHLPP2	215	EGFR	149
HSP90AA1	209	JUN	144
TOP2A	205	HSP90AA1	140
MAPK1	203	PIK3CA	134
JUN	193	ACLY	119

### Functional annotation and pathway enrichment analysis

**Fig. 4:**
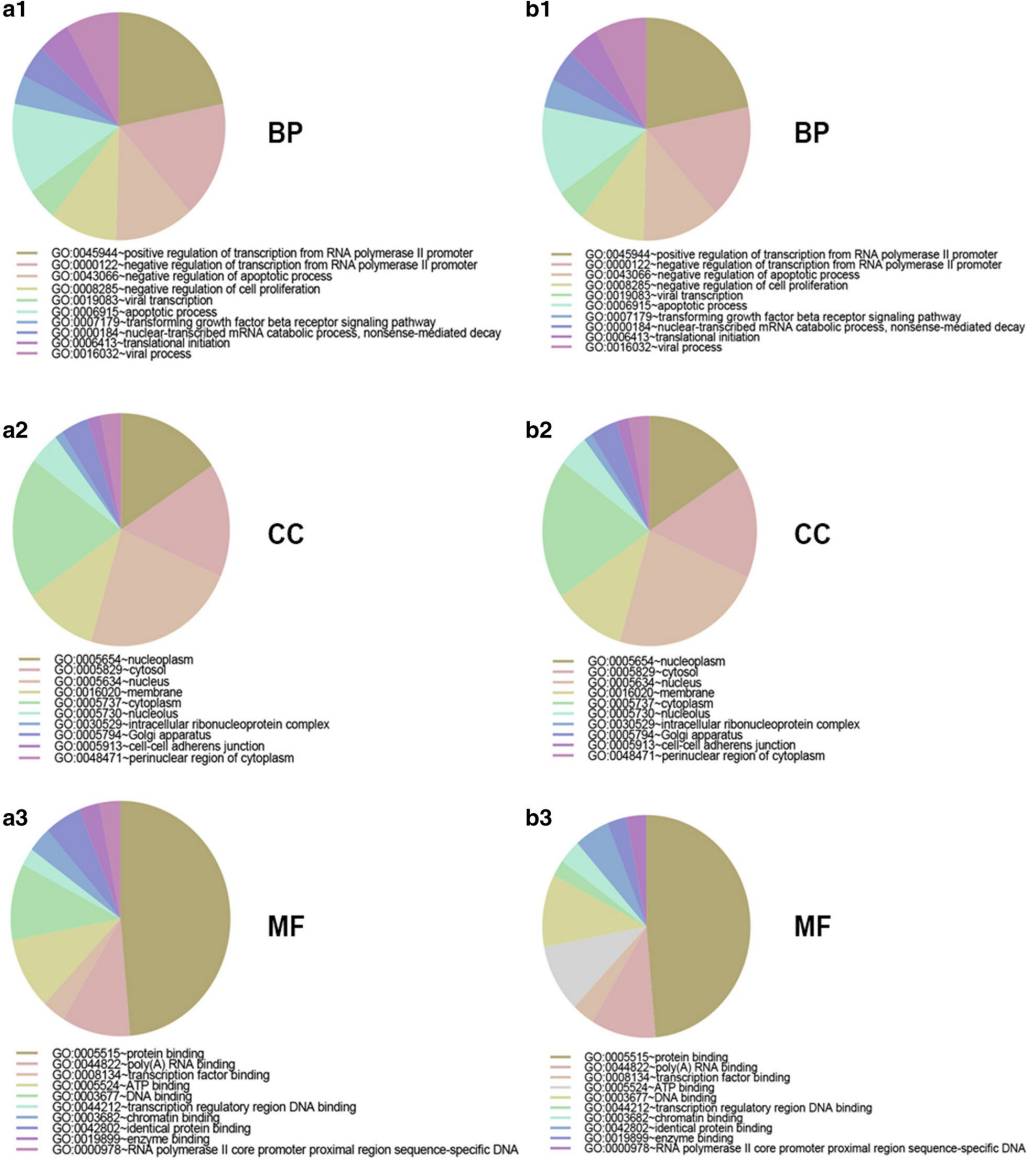
The GO annotation for the predicted target genes of potential DE-miRNAs. **a1** Top 10 enriched biological process (BP) for target genes of upregulated DE-miRNAs; **a2** top 10 enriched cellular component (CC) for target genes of upregulated DE-miRNAs; **a3** top 10 enriched molecular function (MF) for target genes of upregulated DE-miRNAs; **b1** top 10 enriched biological process (BP) for target genes of upregulated DE-miRNAs; **b2** top 10 enriched cellular component (CC) for target genes of upregulated DE-miRNAs; **b3** top 10 enriched molecular function (MF) for target genes of upregulated DE-miRNAs

GO functional annotation was first performed to understand the systematic features and biological meaning of these predicted targets using DAVID database. The GO ontology contains three categories: cellular component (CC), biological process (BP) and molecular function (MF). The top 10 GO terms of targets of upregulated DE-miRNAs were presented in Fig. 4a1–3, including nucleus and cytoplasm in the CC category, transcription DNA-templated and regulation of transcription DNA-templated in the BP category and protein binding and metal ion binding in the MF category. The top 10 GO terms of targets of downregulated DE-miRNAs were shown in Fig. 4b1–3, including nucleus and cytoplasm in the CC category, transcription DNA-templated and positive regulation of transcription from RNA polymerase II promoter in the BP category and protein binding and DNA binding in the MF category.

**Fig. 5:**
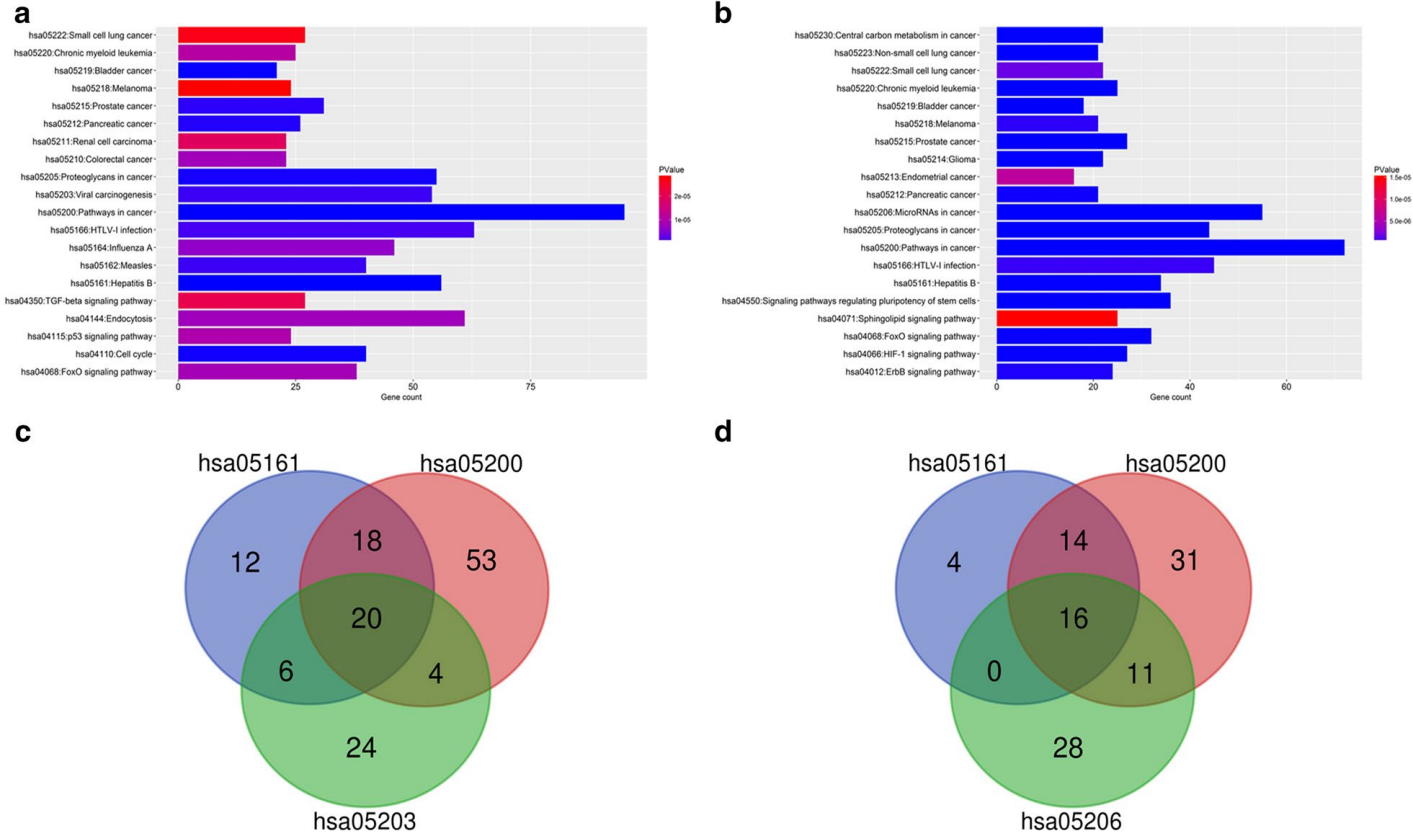
The pathway enrichment analysis for the predicted target genes of potential DE-miRNAs. **a** The top 10 enriched KEGG pathways for target genes of upregulated DE-miRNAs; **b** the top 10 enriched KEGG pathways for target genes of downregulated DE-miRNAs; **c** the intersection of three pathways associated with HBV-related HCC for the target genes of upregulated DE-miRNAs; **d** the intersection of three pathways associated with HBV-related HCC for the target genes of downregulated DE-miRNAs

KEGG pathway enrichment analysis revealed that the targets of upregulated DE-miRNAs were enriched in pathways in cancer, PI3K-Akt signaling pathway, HTLV-I infection, endocytosis and microRNAs in cancer, whereas the targets of downregulated DE-miRNAs were enriched in pathways in cancer, microRNAs in cancer, PI3K-Akt signaling pathway, HTLV-I infection and proteoglycans in cancer. The top 20 enriched KEGG pathways of targets of upregulated and downregulated DE-miRNAs were presented in Fig. 5a, b, respectively.

### Screening of candidate genes in HBV-related HCC

In this study, we intended to identify these miRNA–mRNA regulatory pathways involved in the onset and progression of HBV-related HCC. Thus, we selected several possibly related pathways from the results of KEGG pathway enrichment analysis for further analysis. For upregulated DE-miRNAs, three pathways including hepatitis B, pathways in cancer and viral carcinogenesis were chosen. The genes commonly appeared in all the three pathways were identified as candidate target genes for further analysis. Similarly, only the same targets of downregulated DE-miRNAs appeared in Hepatitis B, pathways in cancer and microRNAs in cancer were screened as candidate genes. Finally, as shown in Fig. 5c, d, 20 and 16 genes were selected as candidate target genes for upregulated and downregulated DE-miRNAs, respectively. For better visualization, miRNA–mRNA networks of these DE-miRNAs and relevant targets were constructed using Cytoscape software (Additional file 3: Figure S2).

It is widely acknowledged that there is an inverse relationship between miRNA expression and target gene expression [27]. Based on this theory, the expression levels of 36 potential target genes in HCC were analyzed using two public databases. After being compared with normal samples, as presented in Additional file 4: Figure S3, only expression of *JUN*, *PIK3R1* and *STAT3* for upregulated DE-miRNAs was significantly decreased in HCC samples. For target genes of downregulated DE-miRNAs, only *E2F2*, *E2F3* and *NRAS* expression was markedly upregulated. The similar results were acquired in our subsequent analysis of expression of *JUN*, *PIK3R1*, *STAT3*, *E2F2*, *E2F3* and *NRAS* in HCC using another database, UALCAN database, and were shown in Additional file 5: Figure S4. According to these results, finally, *JUN*, *PIK3R1*, *STAT3, E2F2*, *E2F3* and *NRAS* were identified as the potential target genes.

### Identification of several potential miRNA–mRNA regulatory pathways in HBV-related HCC

**Fig. 6:**
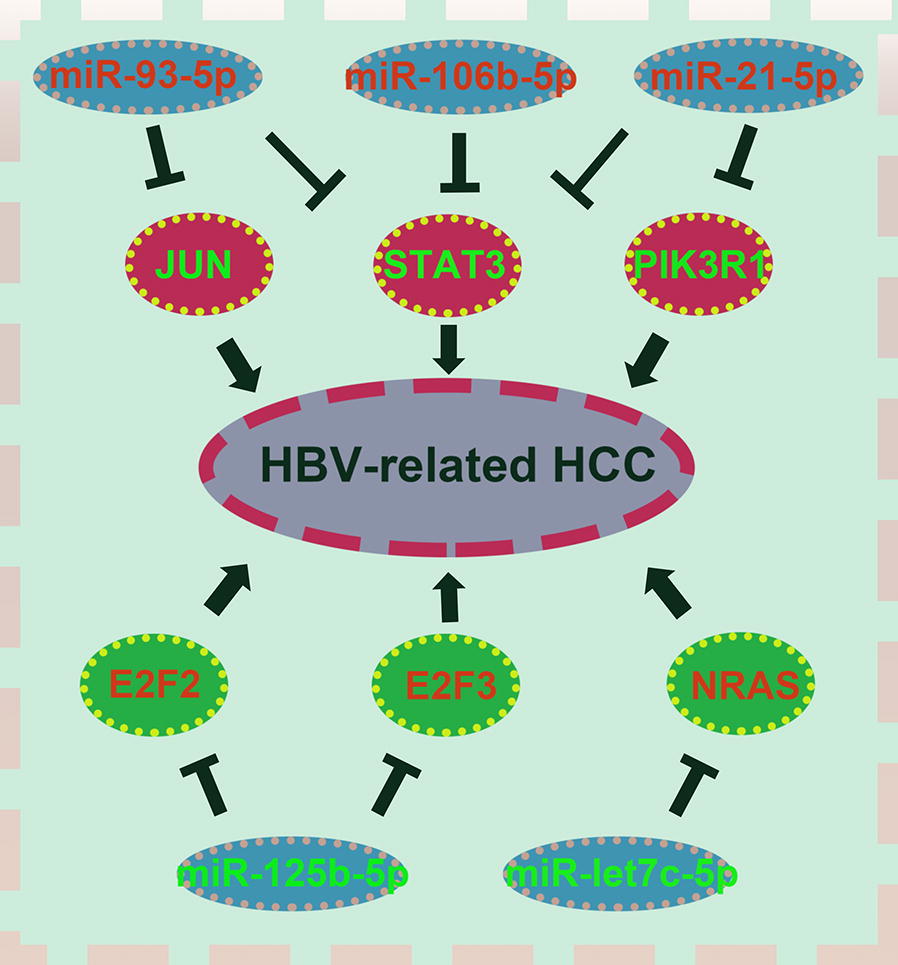
Identified potential miRNA–mRNA regulatory network contributing to pathogenesis of HBV-related HCC

Potential DE-miRNAs and target genes have been identified based on a series of bioinformatic analyses. By matching miRNA–mRNA pairs in miRNet database, we established a potential miRNA–mRNA regulatory network (miR-93-5p-JUN/STAT3 pathway, miR-106b-5p-STAT3 pathway, miR-21-5p-STAT3/PIK3R1 pathway, miR-125b-5p-E2F2/E2F3 pathway and miR-let7c-5p-NRAS pathway) as depicted in Fig. 6.

**Fig. 7:**
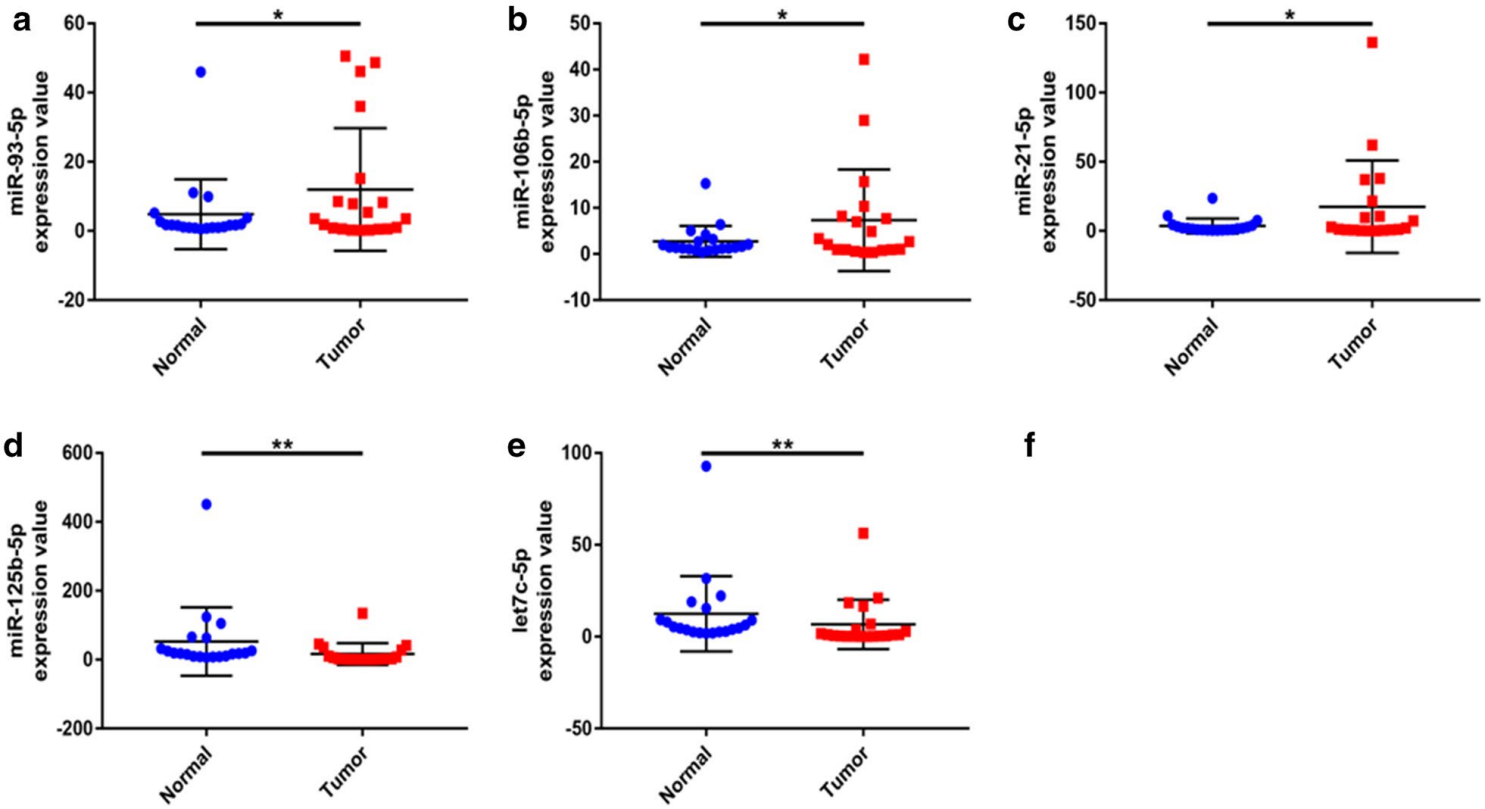
The expression levels of five potential DE-miRNAs in clinical HBV-related HCC tissues compared to matched normal tissues. **a** For miR-93-5p; **b** for miR-106b-5p; **c** for miR-21-5p; **d** for miR-125b-5p; **e** for let7c-5p. *< 0.05; **< 0.01

**Fig. 8:**
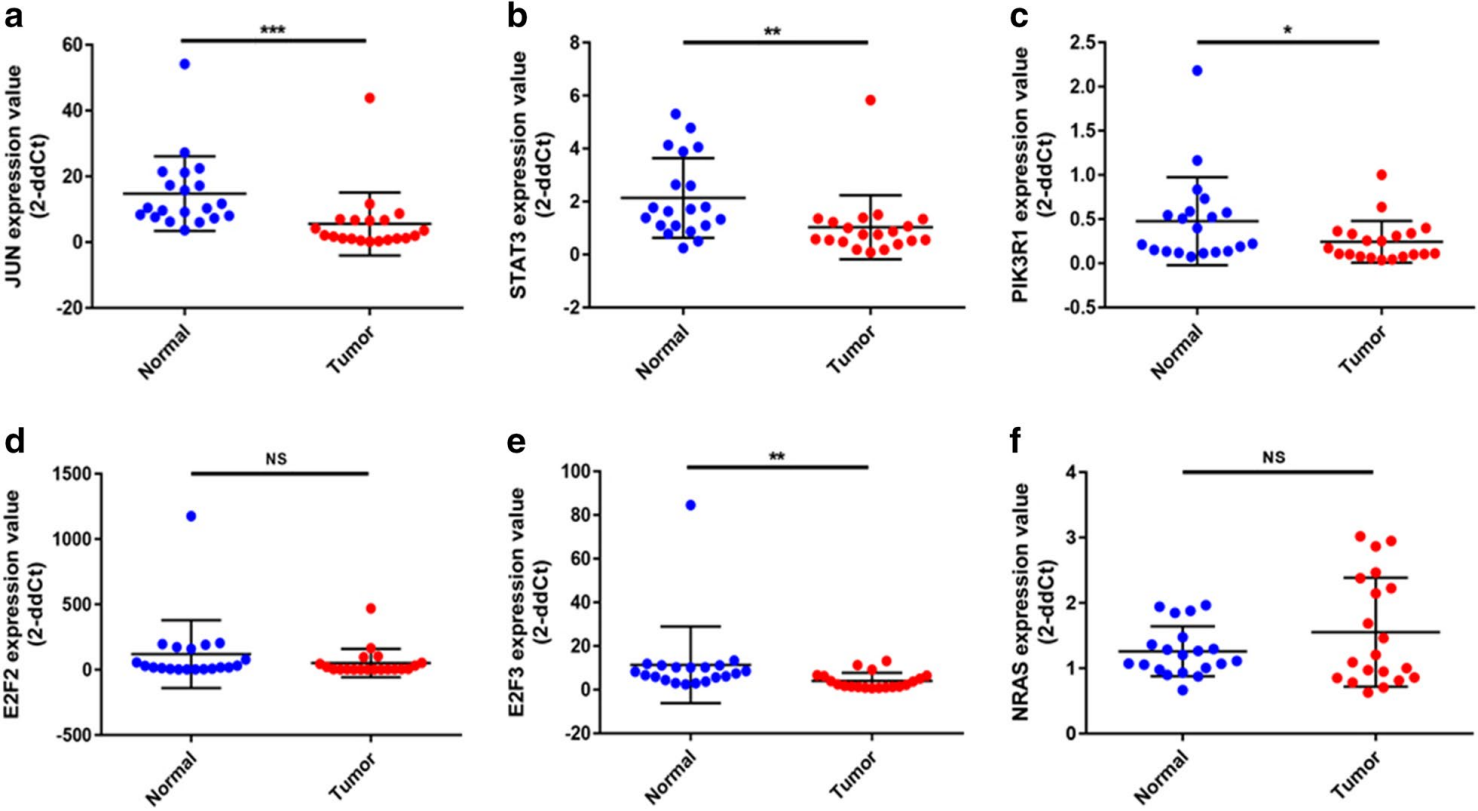
The expression levels of six potential target genes in clinical HBV-related HCC tissues compared to matched normal tissues. **a** For JUN; **b** for STAT3; **c** for PIK3R1; **d** for E2F2; **e** for E2F3; **f** for NRAS. *< 0.05; **< 0.01; ***< 0.001; NS represents no significance

**Fig. 9:**
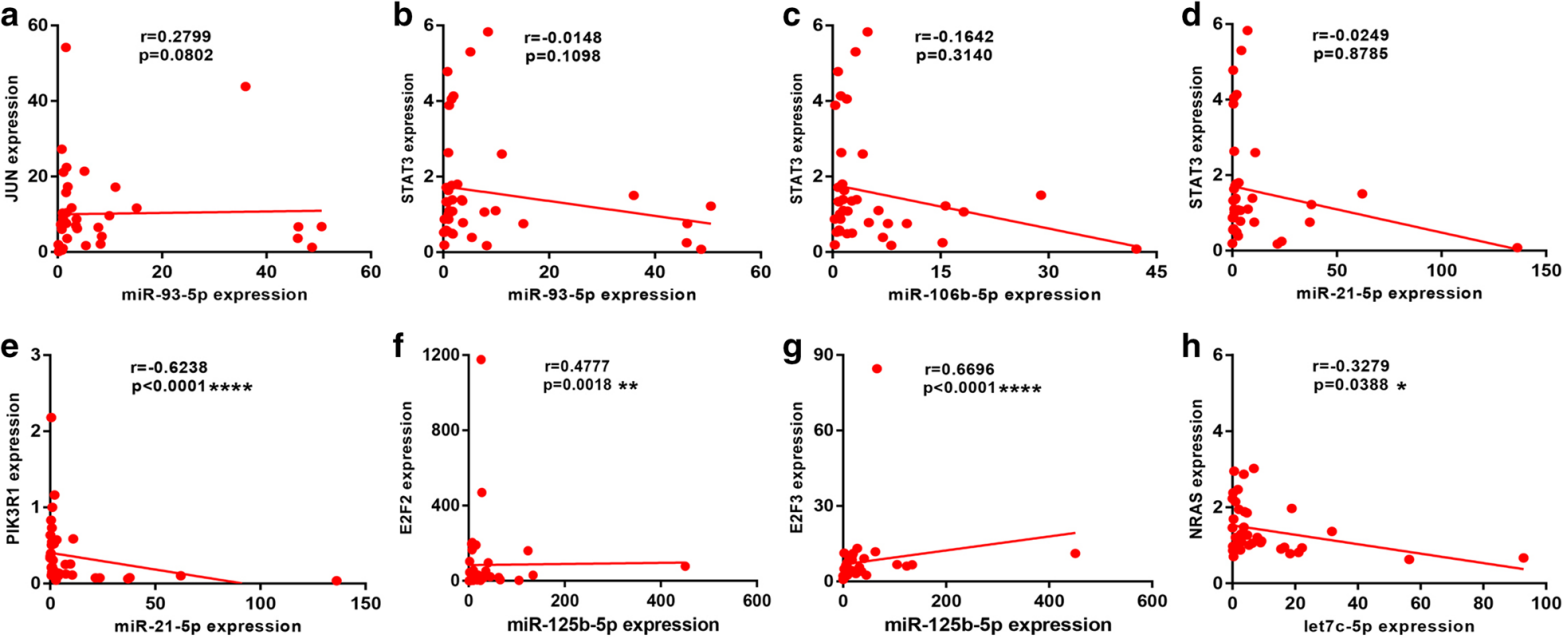
The correlation of potential DE-miRNAs and target genes. **a** For JUN and miR-93-5p; **b** for STAT3 and miR-93-5p; **c** for STAT3 and miR-106b-5p; **d** for STAT3 and miR-21-5p; **e** for PIK3R1 and miR-21-5p; **f** for E2F2 and miR-125b-5p; **g** for E2F3 and miR-125b-5p; **h** for NRAS and let7c-5p

**Fig. 10:**
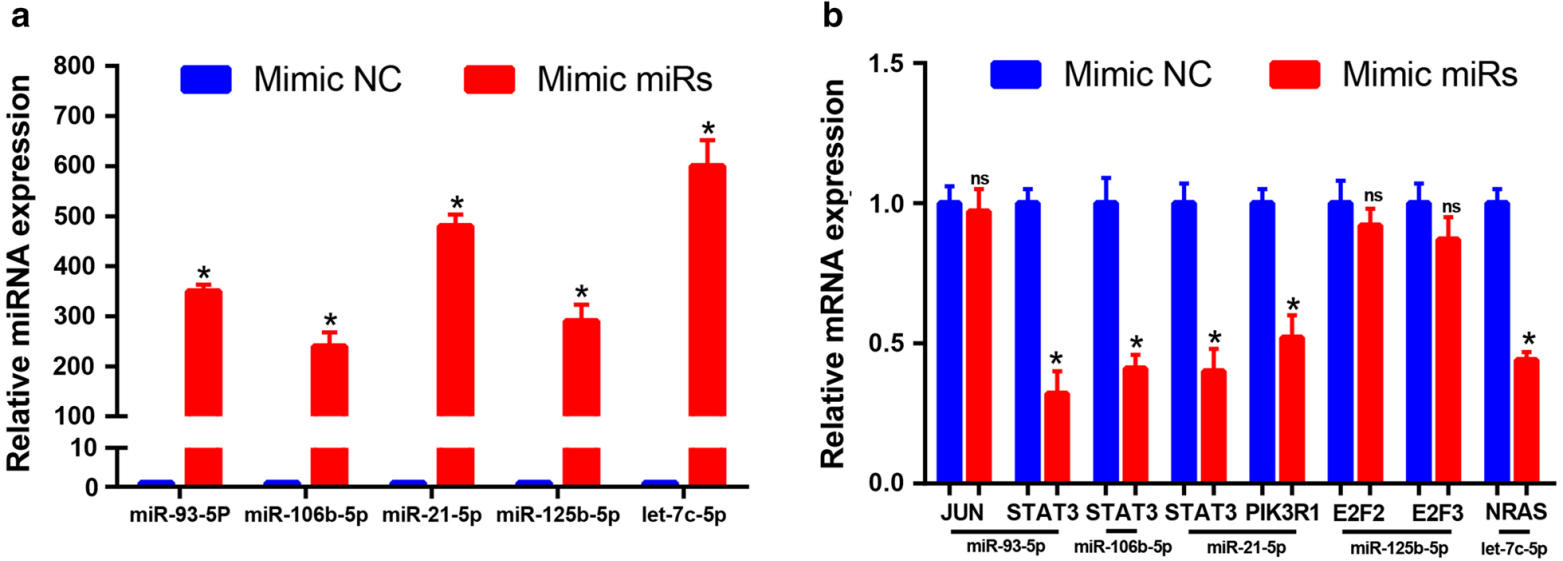
The expression levels of target genes after transfection of the potential miRNA mimics. **a** Hep3B transfected with miRNA mimics exhibited higher expression of miRNAs than mimic negative control; **b** the alteration of target gene expression after transfection of miR-93-5p, miR-106b-5p, miR-21-5p, miR-125b-5p and let7c-5p mimics analyzed in Hep3B cell line using qRT-PCR. *< 0.05

To validate these pathways, the expression levels of these DE-miRNAs and target genes were further detected in 20 pairs of HBV-related HCC clinical tissue samples and matched adjacent normal liver tissues. As shown in Fig. 7, in accordance with our previous analytic results, miR-93-5p, miR-106b-5p and miR-21-5p were significantly upregulated whereas miR-125b-5p and let7c-5p were markedly downregulated in HBV-related HCC compared to matched normal liver samples. Next, the expression levels of six potential targets were determined and the results were presented in Fig. 8. Among the six target genes, when compared with normal liver samples, JUN, STAT3 and PIK3R1 expression were significantly decreased in HBV-related HCC samples. Regarding to E2F2 expression, there was no significant difference between cancer tissues and normal controls. Different from the result of bioinformatics analysis, E2F3 expression were significantly downregulated in HBV-related HCC tissues. Despite no statistical significance, the expression of NRAS was markedly higher in HBV-related HCC tissues than that in matched normal liver tissues. Subsequently, we further analyzed the correlation of miRNA expression and target expression. As shown in Fig. 9, identical with our previous in silico analysis, STAT3 expression was inversely linked to the expression of miR-93-5p, miR-106b-5p and miR-21-5p. A significant negative correlation was found between PIK3R1 expression and miR-21-5p expression. Besides, we observed that NRAS expression was also conversely associated with let7c-5p expression. However, positive correlations of JUN and miR-93-5p, miR-125b-5p and E2F2 and miR-125b-5p and E2F3 were found. Transfected with the five miRNAs mimics, we found that the expression levels of these miRNAs were significantly increased (Fig. 10a). Overexpression of miR-93-5p, miR-106b-5p and miR-21-5p could distinctly attenuate STAT3 expression (Fig. 10b). When compared with negative control groups, downregulation of PIK3R1 and NRAS was also discovered in miR-21-5p and let7c-5p overexpression groups, respectively (Fig. 10b). However, no significant decrease of JUN, E2F2 and E2F3 expression were observed in Hep3B cells transfected with miR-93-5p and miR-125b-5p mimics. All these findings suggested that miR-93-5p/miR-106b-5p/miR-21-5p-STAT3, miR-21-5p-PIK3R1 and let7c-5p-NRAS may fuel the pathogenesis of HBV-related HCC.

## Discussion

The functional pattern of miRNA–mRNA regulatory network has been shown in the onset and progression of a variety of human diseases including cancer [28–30]. Moreover, a lot of studies about miRNA and mRNA expression profile in HBV-related HCC were published [19, 31–34]. However, a comprehensive miRNA–mRNA regulatory network in HBV-related HCC remains largely insufficient. In this present work, we aimed to construct the potential miRNA–mRNA regulatory network in HBV-related HCC. By the way of a series of bioinformatic analyses and experimental validation, several miRNAs, target genes and miRNA–mRNA regulatory pathways which may be involved in the development of HBV-related HCC have been identified.

Two regulatory pathways, miR-93-5p-JUN and miR-106b-5p-STAT3, are identified to contribute to the tumorigenesis of HBV-related HCC and miR-25 is also found to be upregulated in HBV-related HCC tissues than the matched normal liver tissues. MiR-106b, miR-93 and miR-25 belong to the miR-106b-25 cluster [35]. Numerous studies have proved that miR-93-5p, miR-106b-5p and miR-25 function as oncogenic miRNAs in HCC. For example, exosomal miR-93-5p enhances proliferation and invasion of HCC via suppression of TIMP2/TP53INP1/CDKN1A [36]; miR-93-5p promotes HCC proliferation and epithelial–mesenchymal transition (EMT)-mediated invasion and metastasis by directly targeting PDCD4 [37]. MiR-106b-5p activates the Wnt/β-catenin pathway by downregulating APC, thereby resulting in the enhancement of HCC proliferation [38]; upregulation of miR-106b-5p enhances migration and metastasis of HCC via the activation of EMT [39]. As for miR-25, it has been reported to promote HCC growth, migration and invasion through directly targeting RhoGDI1 [40]. Besides, miR-106b-25 cluster has already been investigated about its association with HBV-related HCC [41, 42]. All these findings support our analytic results that miR-93-5p/miR-106b-5p-STAT3 pathways may contribute to the progression of HBV-related HCC.

MiR-21-5p-PIK3R1 pathway is an oncogenic axis in HBV-related HCC in our present work. Previous studies have also shown that miR-21 can boost development of HCC [43–46]. MiR-21 can be modulated HBx, and is involved in the inhibition of apoptosis in HCC by directly targeting IL-12 [47]. However, an opposite result was acquired in another report published by the team of Bandopadhyay [48]. They indicated that the expression of miR-21 was negatively modulated by HBx protein. This contradiction makes certification of expression and function of miR-21 in HBV-related HCC sense. Future experimental validation of our current analytic result—the promotion of miR-21-5p-PIK3R1 pathway in carcinogenesis of HBV-related HCC—is of great significance.

Our analysis suggested that let7c-5p-NRAS and miR-125b-5p-E2F2/E2F3 are several tumor suppressive pathways in HBV-related HCC. Plenty of investigations have verified that let7c-5p hinders progression of HCC. For example, let7c-5p and miR-199a-5p cooperatively suppress migration and invasion in HCC by inhibiting MAP4K3 [49]; let7c-5p inhibits HCC cell proliferation and induces cell cycle arrest via the direct inhibition of CDC25A [50]. MiR-125b-5p is also shown to function as tumor suppressor in HCC. MiR-125b-5p attenuates HCC proliferation via inhibition of Sirtuin 7 [51]; miR-125b-5p suppresses HCC malignancy through targeting SIRT6 [52]. Besides, miR-125b-5p can also be served as a diagnostic biomarker of HBV-related HCC in early stage. These reports mentioned above all hint tumor suppressive roles of let7c-5p-NRAS, miR-125b-5p-E2F2 and miR-125b-5p-E2F3 in HBV-related HCC.

Subsequent experiments further supported our previous in silico analysis. Based on the present experimental results, among all identified miRNA–mRNA regulatory pathways, miR-93-5p/miR-106-5p/miR-21-5p-STAT3, miR-21-5p-PIK3R1 and let7c-5p-NRAS axes demonstrated better prospects. In the future, more emphases need to be put on these regulatory pathways.

Although we performed a comprehensive analysis and experimental validation of miRNA–mRNA regulatory network involved in HBV-related HCC and successfully identify several potential miRNA–mRNA pathways which may be possibly linked to development of HBV-related HCC, some limitations were existed in this study: (1) the sample size of the microarray used here was not large enough, only containing 10 tissue samples; (2) the multilayers of assumption including in silico identification of DE-miRNAs, search for their potential targets, analysis of the potential targets and evaluating the relationship between miRNAs and targets lacked experimental confirmation in vitro and in vivo; (3) experimental validation of potential miRNA and mRNA expression was only conducted in 20 pairs of HBV-related HCC tissues and matched normal tissues. So, future investigations with larger clinical samples and corresponding experiments are required and need to be urgently launched.

## Conclusions

In summary, our present in silico analyses and preliminary experimental validation indicate several potential miRNA–mRNA pathways contributing to the carcinogenesis of HBV-related HCC. We hope that these findings we presented are helpful for future in-depth studies and accelerate the process of overcoming HBV-related HCC.

## Additional files

**Additional file 1: Figure S1.** Normalization of GSE69580. (A) Data before normalization; (B) data after normalization.

**Additional file 2: Table S1.** Primers sequence list.

**Additional file 3: Figure S2.** The miRNA-hub gene networks. (A) For upregulated DE-miRNAs; (B) for downregulated DE-miRNAs.

**Additional file 4: Figure S3.** The expression levels of screened potential target genes from the Oncomine database.

**Additional file 5: Figure S4.** The expression levels of six screened target genes from the UALCAN database.

## Abbreviations

BP: biological process
CC: cellular component
cDNA: complementary DNA
CI: confidence interval
DE-miRNA: differentially expressed miRNA
EMT: Epithelial–mesenchymal transition
GEO: Gene Expression Omnibus
GO: Gene Ontology
HR: hazard ratio
HBV: hepatitis B virus
HBx: HBV X protein
HCC: hepatocellular carcinoma
MiRNA: microRNA
MF: molecular function
KM plotter: Kaplan–Meier plotter
KEGG: Kyoto Encyclopedia of Genes and Genomes
PPI: protein–protein interaction
SD: standard deviation
