# Supplementary figures and images for: Identification of potential miRNA–mRNA regulatory network contributing to pathogenesis of HBV-related HCC
Source: J Transl Med. 2019 Jan 3;17:7. doi: 10.1186/s12967-018-1761-7 (PMC6317219; doi:10.1186/s12967-018-1761-7)

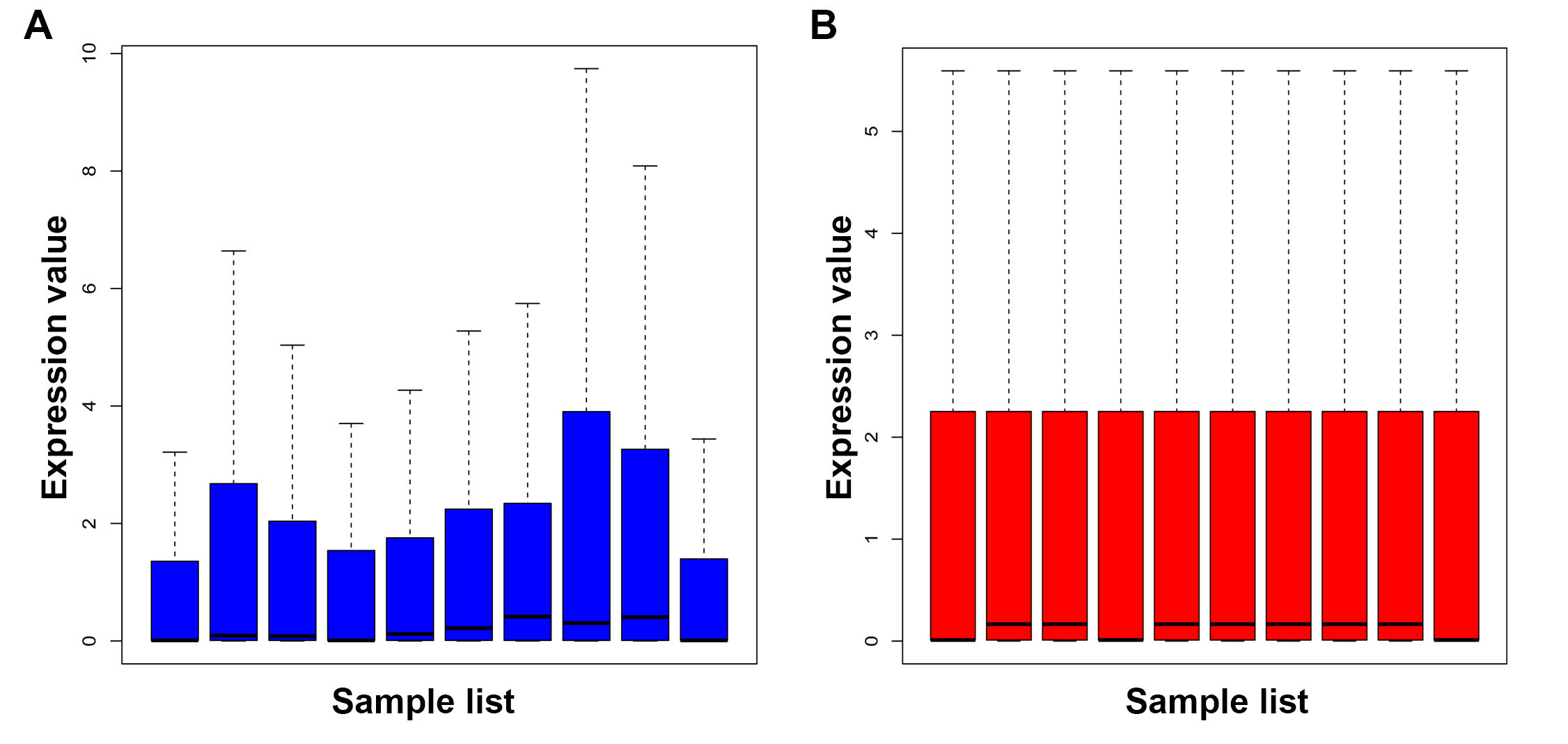

Supplement: Supplementary file 1 — Additional file 1: Figure S1. Normalization of GSE69580. (A) Data before normalization; (B) data after normalization. [file 12967_2018_1761_MOESM1_ESM.jpg]

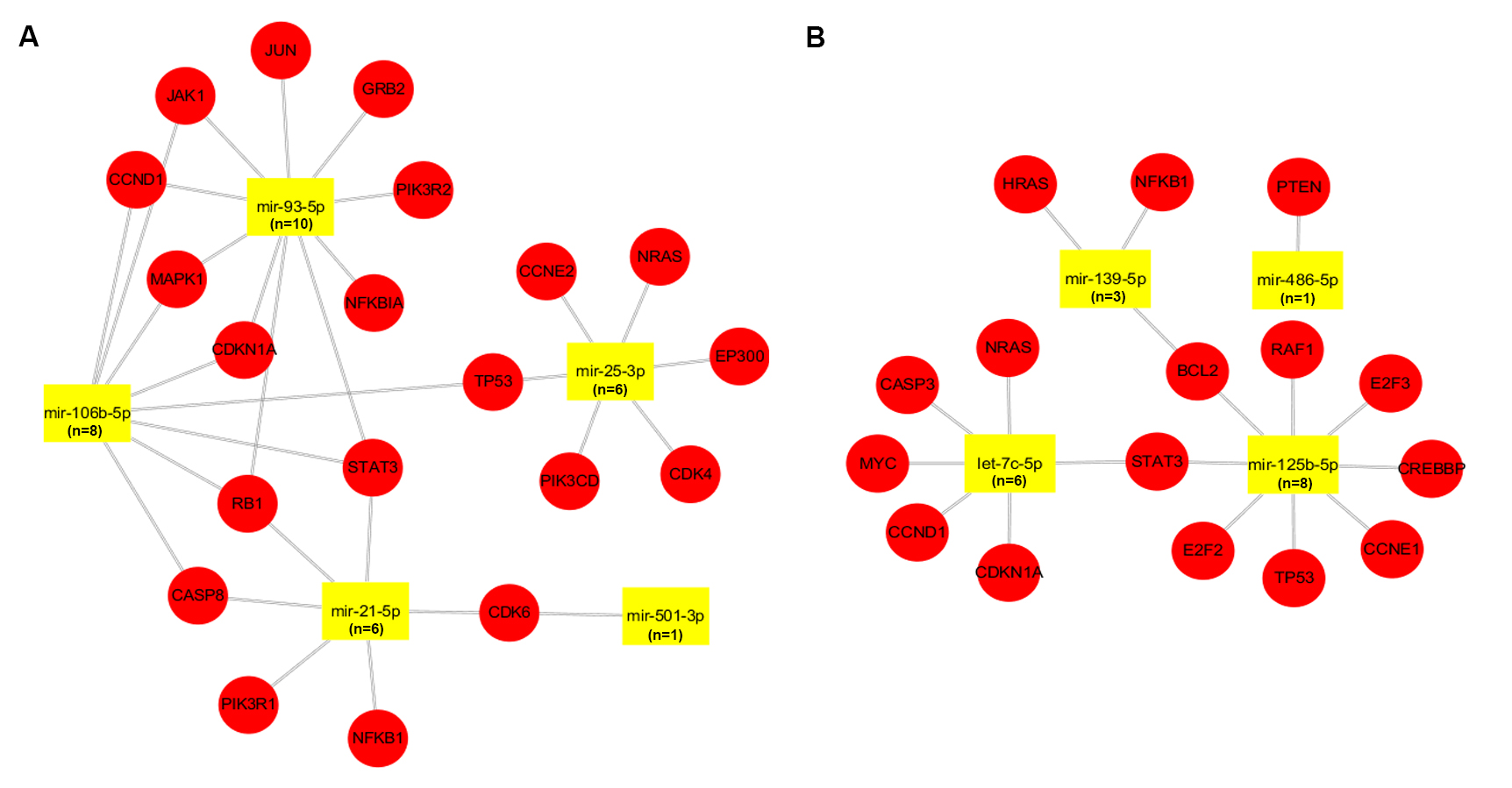

Supplement: Supplementary file 3 — Additional file 3: Figure S2. The miRNA-hub gene networks. (A) For upregulated DE-miRNAs; (B) for downregulated DE-miRNAs. [file 12967_2018_1761_MOESM3_ESM.jpg]

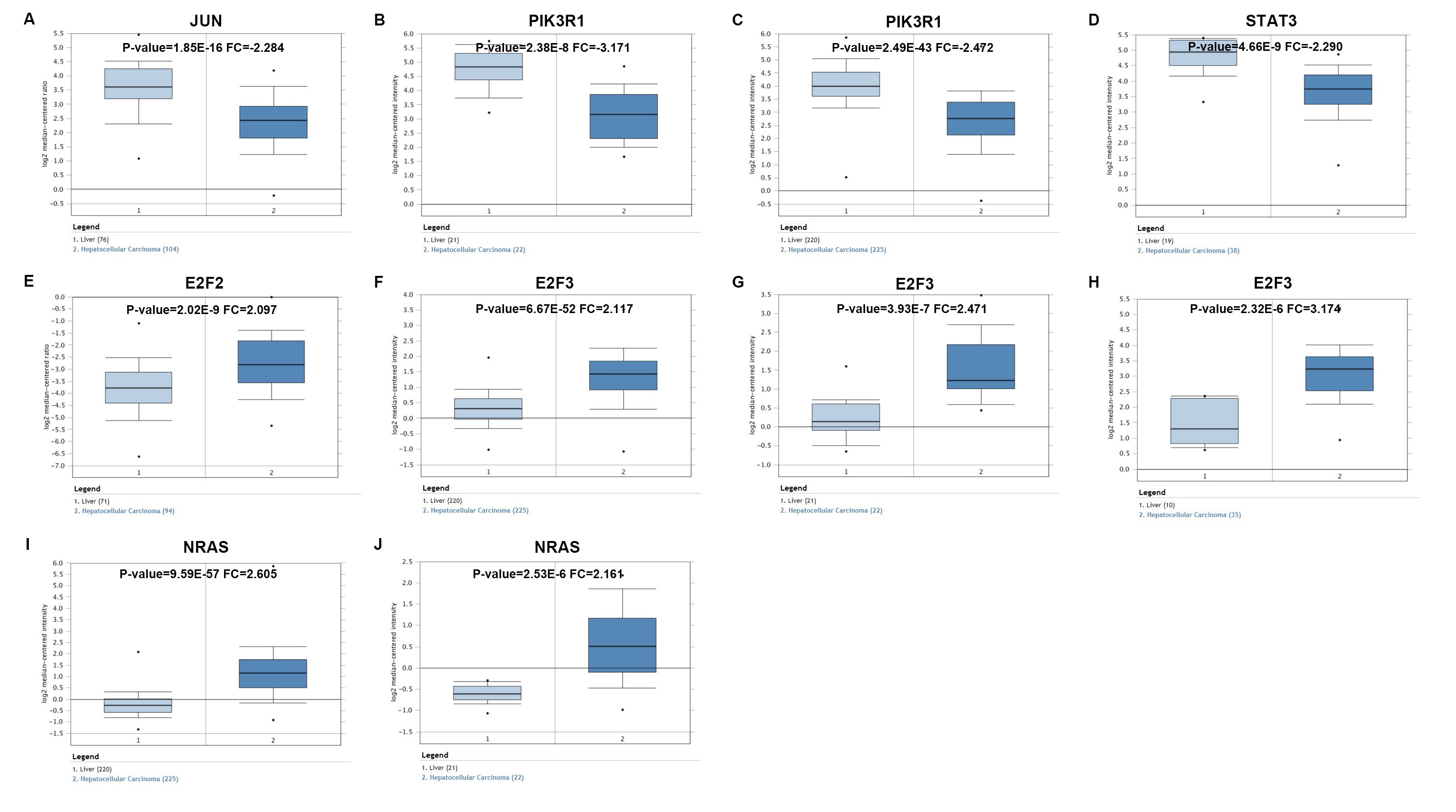

Supplement: Supplementary file 4 — Additional file 4: Figure S3. The expression levels of screened potential target genes from the Oncomine database. [file 12967_2018_1761_MOESM4_ESM.jpg]

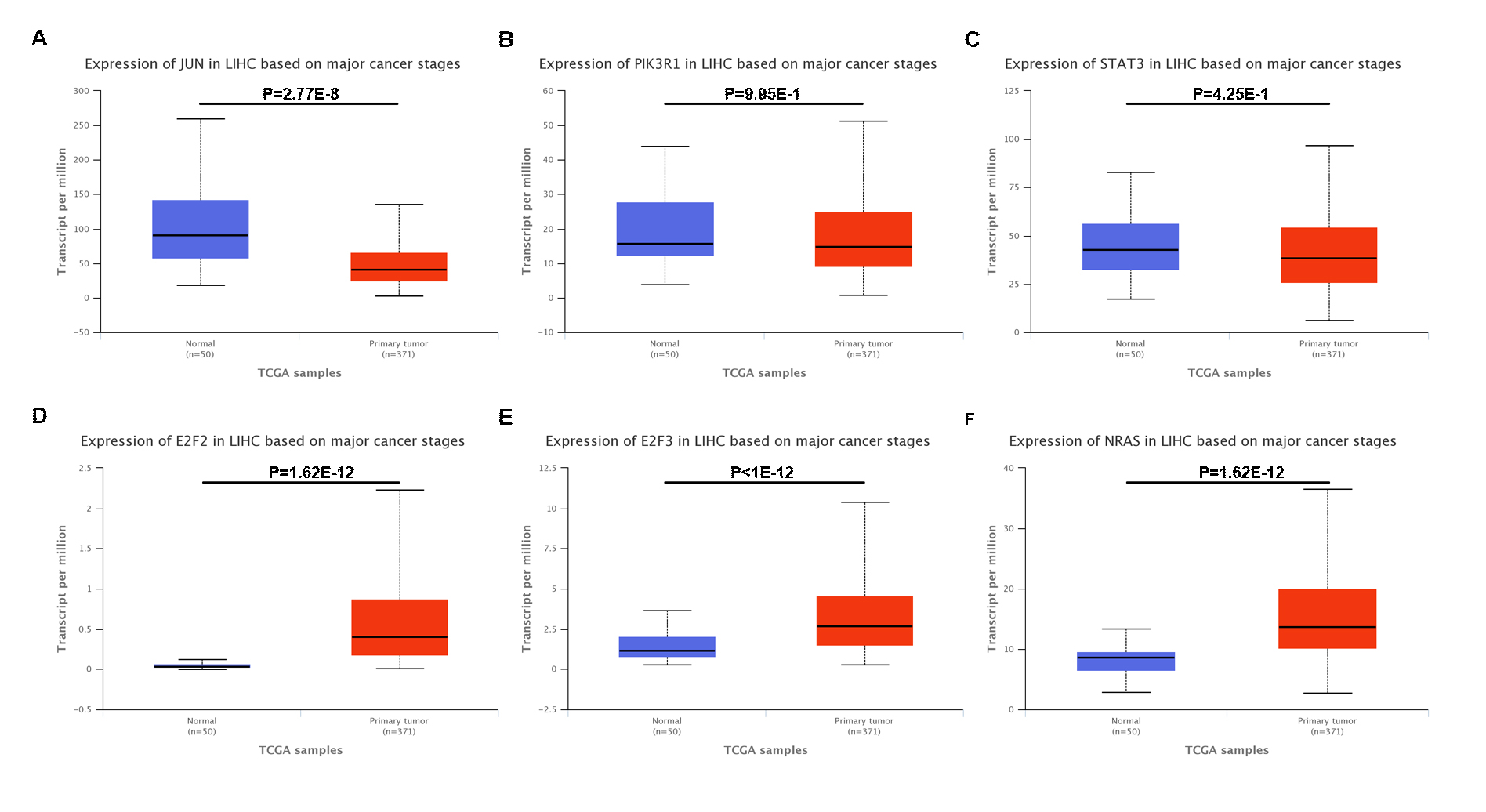

Supplement: Supplementary file 5 — Additional file 5: Figure S4. The expression levels of six screened target genes from the UALCAN database. [file 12967_2018_1761_MOESM5_ESM.jpg]
