# Supplementary material for: Identification of potential miRNA–mRNA regulatory network contributing to pathogenesis of HBV-related HCC
Source: J Transl Med. 2019 Jan 3;17:7. doi: 10.1186/s12967-018-1761-7 (PMC6317219; doi:10.1186/s12967-018-1761-7)
Supplement: Supplementary file 2 — Additional file 2: Table S1. Primers sequence list. [file 12967_2018_1761_MOESM2_ESM.docx]

Table S1. Primers sequence list.

| Protein-coding genes | Primer sequences |
| --- | --- |
| GAPDH-F | TGCACCACCAACTGCTTAGC |
| GAPDH-R | GGCATGGACTGTGGTCATGAG |
| JUN-F | TCCAAGTGCCGAAAAAGGAAG |
| JUN-R | CGAGTTCTGAGCTTTCAAGGT |
| STAT3-F | ACCAGCAGTATAGCCGCTTC |
| STAT3-R | GCCACAATCCGGGCAATCT |
| PIK3R1-F | TGGACGGCGAAGTAAAGCATT |
| PIK3R2-R | AGTGTGACATTGAGGGAGTCG |
| E2F2-F | CGTCCCTGAGTTCCCAACC |
| E2F2-R | GCGAAGTGTCATACCGAGTCTT |
| E2F3-F | AGAAAGCGGTCATCAGTACCT |
| E2F3-R | TGGACTTCGTAGTGCAGCTCT |
| NRAS-F | TGAGAGACCAATACATGAGGACA |
| NRAS-R | CCCTGTAGAGGTTAATATCCGCA |
